# Clear Cell Sarcoma of the Kidney Case Reports Should Connect Renal‐Origin Imaging With Staging, Treatment Rationale, and Follow‐Up

**DOI:** 10.1002/ccr3.73058

**Published:** 2026-06-29

**Authors:** Muhammad Abdullah Awan, Hammad‐ud‐din Raza

**Affiliations:** ^1^ Wah Medical College, National University of Medical Sciences Rawalpindi Pakistan; ^2^ Wah Medical College Rawalpindi Pakistan

**Keywords:** clear cell sarcoma of the kidney, lymph‐node pathology, metastatic staging, pediatric renal tumor, surveillance, Wilms tumor mimic

## Abstract

In pediatric clear cell sarcoma of the kidney, ultrasound and CT are valuable for identifying renal‐origin malignant features, but imaging alone does not fully communicate oncologic risk. Future case reports should connect imaging and histopathology with staging basis, nodal pathology, margin/spillage status, treatment protocol, radiotherapy rationale, response assessment, and surveillance planning.

Dear Editor,

1

We read with great interest the case report by Pedun et al., titled “Clear Cell Sarcoma of the Kidney: A Kidney Malignancy in an 8‐Year‐Old Female Patient,” published in Clinical Case Reports [1]. The authors describe an 8‐year‐old female who presented with a painless right flank mass and was found on ultrasound and contrast‐enhanced CT urogram to have a heterogeneous right renal mass with cystic‐solid components, hydronephrosis, and a claw sign indicating renal origin [1]. Radical nephrectomy was performed, histopathology confirmed clear cell sarcoma of the kidney (CCSK), and chemotherapy was initiated [1]. The report is clinically useful because it reminds readers that CCSK may mimic Wilms tumor and that histopathology remains essential for definitive diagnosis [1].

However, one point deserves stronger emphasis: in CCSK, renal‐origin imaging should be followed by complete oncologic reporting. Pedun et al. state that the tumor was staged as III because of size and nodal involvement, and that the patient was tolerating chemotherapy with ongoing multidisciplinary care [1]. Yet the report does not provide the lymph‐node sampling count, number of positive nodes, margin status, capsular breach, intraoperative rupture or spillage, renal sinus or perinephric fat involvement, exact chemotherapy protocol, radiotherapy decision, or planned surveillance schedule [1]. These details are important because they explain how the diagnosis translated into risk assessment and management.

This distinction matters because the authors themselves emphasize that CCSK differs from Wilms tumor in prognosis and may be associated with metastatic behavior, including bone metastases [1]. Therefore, a case report that focuses mainly on ultrasound, CT urogram, nephrectomy, and histopathologic confirmation may be diagnostically helpful but still incomplete from a pediatric oncology perspective. The CT findings in the report successfully establish that the mass arose from the kidney and caused hydronephrosis [1]. However, renal origin is not the same as full staging. Readers also need to understand whether metastatic evaluation was performed, how nodal involvement was confirmed, and whether the stage III label changed the postoperative treatment plan.

The staging statement would particularly benefit from clarification. The report notes stage III disease due to size and nodal involvement [1], but the basis of nodal involvement is not described in sufficient detail. Future similar reports should specify whether nodes were clinically enlarged, radiologically suspicious, surgically sampled, or histopathologically positive. They should also describe the number and location of sampled nodes, the number involved, and whether surgical margins were free of tumor. Such reporting would make the case more reproducible for clinicians who may encounter a Wilms‐like renal mass but require postoperative information to determine prognosis and referral urgency.

Treatment reporting could also be made more explicit. Pedun et al. mention that chemotherapy was initiated, and the introduction notes that management may involve radical nephrectomy, chemotherapy, and radiation [1]. However, the case section does not state the chemotherapy regimen, number of planned cycles, whether radiotherapy was considered, or why radiotherapy was given or omitted [1]. In a rare tumor report, these details do not merely add length; they convert a diagnostic case into a practical clinical lesson, especially for readers in settings where pediatric oncology protocols and referral pathways may vary.

Follow‐up is another important area. The authors appropriately acknowledge lack of post‐treatment imaging to assess response as a limitation [1]. This limitation could be developed into a broader reporting recommendation. Future CCSK case reports should include planned post‐nephrectomy imaging, response assessment during chemotherapy, renal function monitoring after nephrectomy, and long‐term surveillance for recurrence or metastasis. If such follow‐up is unavailable at the time of publication, stating the planned surveillance pathway would still improve the usefulness of the case for clinicians and families.

Our comments are not intended to reduce the value of the authors' report. Pedun et al. provide a clear and educational radiologic description of a rare pediatric renal malignancy and appropriately highlight that CCSK can resemble Wilms tumor [1]. Rather, the broader lesson is that CCSK reporting should connect three components: first, imaging evidence of renal origin; second, pathological and surgical details that justify staging; and third, treatment and follow‐up information that explains how risk is managed after diagnosis.

Overall, this case contributes to pediatric renal tumor awareness, particularly in resource‐limited settings where early ultrasound and CT may guide timely referral and surgical planning [1]. Its clinical value would be even stronger if future reports used a structured oncologic dataset: imaging findings, histopathologic confirmation, nodal details, margin/spillage status, treatment protocol, radiotherapy rationale, response assessment, and surveillance plan. This would allow readers to move from recognizing CCSK to understanding how it should be staged, treated, and followed.

## 2 Conclusion

Pedun et al. present an important case of CCSK in an 8‐year‐old female and effectively show its radiologic similarity to Wilms tumor [1]. However, future pediatric CCSK case reports should move beyond renal‐origin imaging and histopathologic diagnosis to include staging basis, nodal pathology, margin and spillage status, treatment protocol, radiotherapy rationale, response imaging, and surveillance planning. Such reporting would make rare tumor case reports more clinically reproducible and more useful for pediatric oncology decision‐making.

## Author Contributions

**Muhammad Abdullah Awan:** conceptualization, data curation, formal analysis, project administration, writing – original draft, writing – review and editing. **Hammad‐ud‐din Raza:** data curation, writing – original draft, writing – review and editing.

## Funding

The authors have nothing to report.

## Ethics Statement

The authors have nothing to report.

## Consent

The authors have nothing to report.

## Conflicts of Interest

The authors declare no conflicts of interest.

## Data Availability

Data sharing not applicable to this article as no datasets were generated or analysed during the current study.
